# The effect of different direct antivirals on hepatic steatosis in nondiabetic and naïve hepatitis C-infected Egyptian patients

**DOI:** 10.1186/s43162-023-00197-1

**Published:** 2023-02-13

**Authors:** Ahmed El-Ghandour, Tarek Youssif, Wesam Ibrahim, Hoda Ahmed Abdelsattar, Somia Abd elhamid Bawady, Mariam Wagih, Sarah El-Nakeep

**Affiliations:** 1 Hepatology and Gastroenterology Unit, Internal Medicine Department, Faculty of Medicine, Ain Shams University, Cairo, Egypt; 2 Clinical Pathology Department, Faculty of Medicine, Ain Shams University, Cairo, Egypt; 3 Armed Forces Medical Complex Kobry El Qobba, Cairo, Egypt

**Keywords:** Hepatitis C, Steatosis, Direct-acting antivirals, Metabolic profile, Transient elastography

## Abstract

**Background:**

Hepatitis C is associated with metabolic effects and fatty liver disease. The effect of different direct antivirals on the liver steatosis, and the metabolic profile, still needs to be established. The aim of this study is to determine the effect of achieving the sustained virological response after 12 weeks (SVR-12 weeks) with different combinations of direct antiviral drugs, on the hepatic steatosis, and fibrosis presented by laboratory and transient elastography parameters. Our study population is nondiabetic, chronically infected HCV Egyptian patients and naïve to any form of HCV treatment.

**Methods:**

This cohort study was carried on 100 nondiabetic HCV treatment-naïve patients attending the Hepatology Clinic, in the Gastroenterology and Hepatology Department, Ain Shams University, and Kobry El Koba Military Hospital. The patients were divided into four groups according to their treatment regimens as follows: group A: 25 patients who received sofosbuvir (400 mg) and daclatasvir (60 mg) daily for 12 weeks; group B: 25 patients who received sofosbuvir (400 mg) and ledipasvir (90 mg) daily for 12 weeks; group C: 25 patients who received ombitasvir (12.5 mg), paritaprevir (75 mg), and ritonavir (50 mg) daily for 12 weeks; and group D: 25 patients who received sofosbuvir (400 mg) and simeprevir (150 mg) daily for 12 weeks. All patients were subjected to the following investigations: HCV quantitative PCR before and after 12 weeks of treatment, clinical and laboratory metabolic evaluation including alfa-fetoprotein level, thyroid profile assessment, ferritin level, pelvi-abdominal ultrasound, and FibroScan examination.

**Results:**

All patients achieved SVR after 12 weeks. FibroScan median decreased (*P* < 0.001) from 19.29 ± 6.97 kPa at baseline to 14.15 ± 6.48 kPa at SVR12. NAFLD score median increased from 1.88 (1.49–2.22) at baseline to 2.01 (1.61–2.33) after 12 weeks of treatment. The highest level of NAFLD score was in group C, and the lowest was in group B. The BMI mean decreased from 28.31 ± 1.53 at baseline to 28.07 ± 1.52 at SVR12. HbA1C level mean decreased from 5.73 ± 0.23 at baseline to 5.40 ± 0.24 at SVR12. In addition, liver enzymes, cholesterol, triglycerides, APRI score (AST-platelet ratio index), and HBA1C decreased after 12-week treatment with a statistically significant difference, while the mean LDL increased after 12 weeks of treatment.

**Conclusions:**

DAAs affect the metabolic profile of the treated patients. There is a noticed improvement in the FibroScan, NAFLD score, and lipid profile after achieving the SVR-12 weeks. However, LDL is increased after viral cure, mostly due to viral-host molecular interaction.

## Introduction

Egypt has the highest prevalence of hepatitis C virus infection worldwide (6.3% in 2015); 90% of the infected population are infected by genotype 4 [1]. The estimated number of patients needed to treat in Egypt to eliminate HCV infection by 2030 was calculated using a modeling study and found to be 350,000 patients per year [2]. The introduction of oral direct-acting antivirals (DAAs) is considered an important breakthrough in virology in the last decade. DAAs generated a great impact on the prognosis and quality of life of HCV patients [3].

The effect of HCV on the metabolic state is well established in the form of: steatosis, steatohepatitis, and hyperlipidemia. The effect of the DAAs on the metabolic profile following HCV cure is still under extensive research. Theoretically, the insulin resistance state caused by HCV infection should be reversed upon cure; however, the data is controversial [4–6]. Insulin resistance and diabetes are important risk factors in increasing the rapidity of progression of the chronic hepatitis to cirrhosis [7] and lowering the response to antiviral medications [8].

The most recent American Association for the Study of Liver Diseases (AASLD) [9] and European Association for the Study of the Liver (EASL) [10] guidelines have removed sofosbuvir and daclatasvir from the regimens used in HCV treatment. However, they are still the primary treatment in the Egyptian guidelines as stated by The National Committee for Control of Viral Hepatitis in Egypt due to their cost-effectiveness, efficacy, and safety in genotype 4.

Transient elastography results could be inconsistent across patients due to the presence of lower stages of fibrosis (F1–2), steatosis, or high BMI (body mass index) [11]. Thus, caution is advised when interpreting these populations, and further research is needed.

The aim of this study is to evaluate the effect of direct-acting antiviral drugs on hepatic steatosis, metabolic profile and scores, and transient elastography scan (FibroScan) parameters, in treating HCV-naïve chronically infected Egyptian patients, with normal baseline metabolic profile (nondiabetic and normo-lipidemic). Then, we evaluate the patients after 3 months of treatment by direct-acting antivirals and the effect of achieving their sustained virological response (SVR) on the metabolic profile and radiologic parameters.

## Methods

This prospective cohort study was carried in the outpatient virology clinic specialized in treating HCV-infected patients (as a part of the National Campaign for HCV treatment) at the Gastroenterology and Hepatology Department, Ain Shams University, and at Kobry El-Kobba Military Hospital (Armed Forces Medical Complex Kobry El Qobba) between January 2018 and August 2019.

The study included 100 chronically infected HCV patients and naïve to HCV treatment. The patients were diagnosed as HCV infected through the national screening campaign by detecting HCV antibodies by ELISA. Further confirmation of active HCV infection is done by quantitative PCR for HCV RNA (COBAS TaqMan; Roche Diagnostic systems) with a lower limit of detection of 15 IU/ml.

This study protocol acquired the approval of the Ethical Committee of Faculty Medicine, Ain Shams University, Cairo, Egypt. Approval number of the protocol is FMASU-MD (322/2017). Informed written consent was taken from all the patients before inclusion in the study. All methods were carried out in accordance with Helsinki guidelines and regulations.

The exclusion criteria included the following:

1. Patients with previous antiviral treatment of any type
2. Patients with HBV or HIV co-infection
3. Pregnant females
4. Patients with any metabolic comorbidity like diabetes, hyperlipidemia, hypercholesterolemia, hyperthyroidism, or hypothyroidism
5. Patients with any autoimmune disease including autoimmune hepatitis
6. Patients with hemochromatosis, Wilson disease, or bilharziasis
7. Patients with hepatocellular carcinoma or with past history of hepatocellular carcinoma
8. Patients with body mass index greater than 28 kg/m^2^
9. Patients with any history of drug intake known to cause hepatic steatosis (e.g., methotrexate, amiodarone, tamoxifen)

The inclusion criteria are as follows:

1. Patients chronically infected with HCV with anti-HCV-positive antibodies and further confirmed by positive PCR for HCV RNA
2. Patients > 18 years old and < 70 years old
3. Patients with platelet count of > 100,000/mm^3^ and hemoglobin level of > 10 g/dl
4. Patients with normal thyroid, lipid profile, and HbA1C level

We calculated the Child-Pugh score for each patient according to the established clinical formulas.

Patients were divided into 4 groups according to their treatment regimen for HCV as follows:

1. Group A: Twenty-five patients who received sofosbuvir (400 mg) and daclatasvir (60 mg) daily for 12 weeks
2. Group B: Twenty-five patients who received sofosbuvir (400 mg) and ledipasvir (90 mg) daily for 12 weeks
3. Group C: Twenty-five patients who received ombitasvir (12.5 mg), paritaprevir (75 mg), and ritonavir (50 mg) daily for 12 weeks
4. Group D: Twenty-five patients who received sofosbuvir (400 mg) and simeprevir (150 mg) daily for 12 weeks.

All patients are subjected to the following at baseline:

(A). Proper history evaluation: To exclude other causes of chronic hepatitis, any associated comorbidities, drug history of any antiviral drugs, or any drugs that induce hepatic steatosis
(B). Full clinical examination: General examination and local abdominal examination to assess the general signs of liver cell failure (jaundice, palmar erythema, spider nevi, bilateral pitting lower limb edema, ascites, hepatic encephalopathy, etc.), hepatomegaly, and splenomegaly. Other systems were thoroughly examined to exclude comorbidities.
(C). Laboratory investigations are as follows:

1. Routine laboratory investigations including complete blood picture, liver enzymes, liver function tests, kidney function tests, urine analysis, lipid profile, fasting, and 2-h postprandial blood glucose and HbA1C
2. Viral markers including HCV Ab by ELISA, HBs Ag, and HIV antibody test
3. HCV RNA by PCR × (COBAS TaqMan; Roche Diagnostic systems), with a lower limit of detection of 15 IU/ml
4. Alpha-fetoprotein and ceruloplasmin levels in serum
5. Thyroid profile (TSH — free T3 — free T4)
6. Serum ferritin, anti-bilharzial antibody, and CPK were tested.
7. Antinuclear antibodies (ANA) and smooth-muscle antibodies (SMAs)
8. Pregnancy test for married female patients in the child-bearing period

(D). Radiological investigations were as follows:

1. Pelvi-abdominal ultrasound: To report any changes of liver echogenicity, or nodularity, signs of portal hypertension (splenomegaly, dilated portal vein, ascites, etc.), or hepatic focal lesions
2. FibroScan: To assess the degree of hepatic fibrosis before starting treatment

Results are expressed in kilopascal (kPa) that corresponds to the manufacturer’s recommendations and to the median of 10 validated measurements. Liver stiffness values range from 2.5 to 75 kPa [11]. Ten valid measures of liver stiffness are indicated with at least 60% success rate and interquartile range < 30% of the median value with results expressed in kilopascals (KPa). When liver stiffness values range from 2.5 to 7 kPa, then the fibrosis is mild or absent, whereas when values area is above 13 kPa, then the cirrhosis is present [12].

(E). Upper endoscopy

Upper endoscopy (Olympus endoscope LUCERA 260) was done to all patients with splenomegaly, or any sign of portal hypertension to exclude the presence of esophageal, or fundal varices.

(F). PRI score is as follows:

AST to platelet ratio index score (APRI score) was calculated for the patients before starting treatment.

It is calculated as (AST/upper limit of normal range)/platelet count (10ˆ9/L) × 100 [13].

There are several optimal APRI cutoff values for prediction of cirrhosis presented in the literature. A lower threshold of 0.5 and a higher one of 1.5 have been suggested for the identification of significant fibrosis [14].

(G). NAFLD fibrosis score

NAFLD fibrosis score was calculated for the patients before starting treatment.

The score was developed in 2007 [15], where the authors stated that hyperglycemia, albumin, age, body mass index (BMI), platelet count, and aspartate aminotransferase (AST)/alanine aminotransferase (ALT) ratio were the solitary predictors of advanced fibrosis.

After 12 weeks of treatment, all patients of the study were subjected to the following:

(A). Proper clinical examination
(B). Laboratory investigations are as follows:
  1) Complete blood picture, liver enzymes, liver and kidney function tests, fasting and 2-h postprandial blood glucose, lipid profile thyroid profile, and HbA1C
  2) HCV RNA by PCR (COBAS TaqMan; Roche Diagnostic systems), with a lower limit of detection of 15 IU/ml
  3) Alpha-fetoprotein level in serum
(C). Radiological investigations are as follows:

1. Pelvi-abdominal ultrasound: To detect any changes of liver echogenicity, nodularity, or signs of portal hypertension (splenomegaly, dilated portal vein, ascites, etc.)
2. FibroScan: To assess the degree of hepatic fibrosis after treatment

(D). APRI score calculation
(E). NAFLD fibrosis score calculation

### Quantitative PCR for HCV RNA

Serum HCV RNA was measured using the COBAS TaqMan HCV Test v2.0 (Roche Molecular Systems, Pleasanton, CA, USA; lower limit of quantitation (LLOQ) of 15 IU/ml) at baseline, all subsequent study visits during treatment, and posttreatment at weeks 4, 12, and 24. Patients with confirmed HCV RNA < LLOQ at the end of the treatment and posttreatment visits continued until the subsequent posttreatment visits, unless there was a confirmed virologic relapse. On-treatment virologic failure was defined as follows: breakthrough, i.e., confirmed by HCV RNA > LLOQ after having previously had HCV RNA < LLOQ while on treatment; rebound, i.e., confirmed > 1-log10 IU/ml increase in HCV RNA while on treatment; or nonresponse, i.e., HCV RNA persistently > LLOQ through 8 weeks of treatment. Relapse was defined as confirmed HCV RNA > LLOQ during the posttreatment period having achieved HCV RNA < LLOQ at the end of the treatment.

### Statistical methods

Data were collected, revised, coded, and entered to the Statistical Package for Social Science (IBM SPSS) version 23. The quantitative data were presented as mean, standard deviations, and ranges when their distribution found parametric and median with interquartile range (IQR) when their distribution found nonparametric. Also, qualitative variables were presented as number and percentages.

The comparison between groups with qualitative data was done by using chi-square test.

The comparison between two groups with quantitative data and parametric distribution was done by using independent *t*-test while for nonparametric data by using Mann-Whitney test.

The comparison between two paired groups with quantitative data and parametric distribution was done by using paired *t*-test while for nonparametric data by using Wilcoxon rank test.

The comparison between more than two groups with quantitative data and parametric distribution was done by using one-way ANOVA test, followed by post hoc analysis using LSD test, while for nonparametric data by using Kruskal-Wallis test, followed by post hoc analysis using Mann-Whitney test.

Spearman correlation coefficients were used to assess the correlation between two quantitative parameters in the same group.

The confidence interval was set at 95%, and the margin of error accepted was 5%. So, the *P*-value was considered significant according to the following:

- *P*-value > 0.05: Nonsignificant
- *P*-value < 0.05: Significant
- *P*-value < 0.01: Highly significant

## Results

Our study evaluated the effect of direct-acting antiviral drugs on hepatic steatosis in HCV chronically infected naïve Egyptian patients at baseline and after reaching 12 weeks. All the patients were males with a mean age of 50.99 ± 8.746. All the patients achieved SVR12 despite receiving different DAA regimens.

**Fig. 1:**
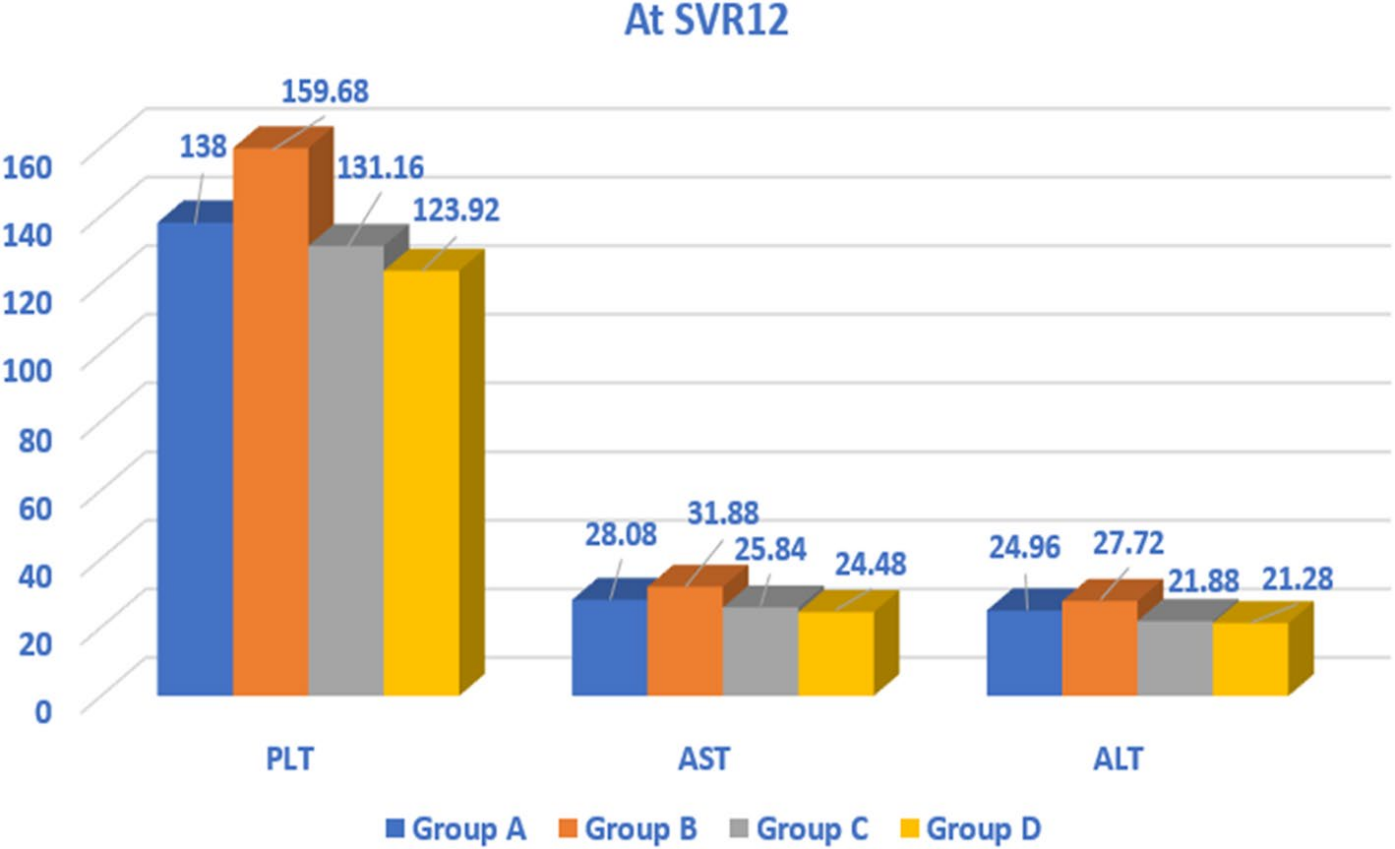
Comparison between the studied groups regarding PLT, AST, and ALT levels at SVR12

The AST, ALT, and platelets were higher in group B at the baseline than the rest of the groups (A, C, and D). The mean PLT count was decreased from baseline upon reaching SVR12. There was difference in the mean PLT count across the four groups; the highest mean PLT count was in group B 159.68 ± 41.72, and the lowest was in group D 123.92 ± 16.01 (Fig. 1).

Liver enzymes (AST and ALT) decreased overtime and upon reaching SVR, where the mean AST was significantly decreased from 58.85±31.85 to 27.57± 9.90 (Table 1). In addition, the mean ALT levels were decreased from 53.54±28.39 at baseline to 23.96±8.92 at SVR12, reflecting a decreased necro-inflammatory activity after treatment. Also, there was a difference in the AST and ALT levels across the four studied groups at SVR12; the highest was in group B, and the lowest was in group D. The levels of serum albumin increased, the total bilirubin decreased, and the AFP decreased upon reaching the SVR12, when compared to the baseline (Tables 1 and 2).

**Table 1:** Comparison between all studied patients’ data before and after treatment

All patients		Before treatment	After treatment (at SVR12)	***p***-value
		No. = 100	No. = 100	
**Hb (gm/dl)**	Mean ± SD Range	13.90 ± 1.11 11.3–15.7	13.05 ± 1.11 10.5–15	0.000
**WBC (× 10^3/μl)**	Mean ± SD Range	6.89 ± 1.67 3.9–11.7	6.42 ± 1.56 4–11	0.000
**PLT (× 10^3/μl)**	Mean ± SD Range	144.14 ± 31.28 108–260	138.19 ± 31.35 95–256	0.000
**AST (U/l)**	Mean ± SD	58.85 ± 31.85	27.57 ± 9.90	0.000
	Range	20–124	14–52	
**ALT (U/l)**	Mean ± SD	53.54 ± 28.39	23.96 ± 8.92	0.000
	Range	15–115	12–48	
**S. alb. (g/dl)**	Mean ± SD	3.88 ± 0.40	4.28 ± 0.40	0.000
	Range	3.2–4.9	3.6–5.3	
**INR**	Mean ± SD	1.02 ± 0.14	1.00 ± 0.06	0.120
	Range	0.1–1.2	0.9–1.1	
**T. bil. (mg/dl)**	Mean ± SD	0.87 ± 0.29	0.63 ± 0.18	0.000
	Range	0.5–1.6	0.4–1.2	
**D. bil. (mg/dl)**	Median (IQR)	0.2 (0.1–0.4)	0.07 (0.03–0.2)	0.000
	Range	0.03–0.9	0.01–0.6	
**BMI**	Mean ± SD	28.31 ± 1.53	28.07 ± 1.52	0.000
	Range	24–31	23.5–30.5	
**FBS (mg/dl)**	Mean ± SD Range	120.26 ± 5.09 108–125	110.70 ± 10.68 13–120	0.000

**Table 2:** Comparison between all studied patients’ data before and after treatment

All patients		Before treatment	After treatment (at SVR12)	***p***-value
		No. = 100	No. = 100	
**TG (mg/dl)**	Mean ± SD	101.68 ± 29.85	88.64 ± 26.53	0.000
	Range	53–199	17–145	
**S. chol. (mg/dl)**	Mean ± SD	124.17 ± 38.55	114.25 ± 35.21	0.000
	Range	55–199	50–188	
**HDL (mg/dl)**	Mean ± SD	36.15 ± 9.63	48.97± 5.63	0.000
	Range	20–55	32–70	
**LDL (mg/dl)**	Mean ± SD	81.79 ± 21.43	88.09 ± 21.80	0.000
	Range	44–136	32–141	
**s.cr. (mg/dl)**	Mean ± SD	0.89 ± 0.13	0.94 ± 0.15	0.000
	Range	0.7–1.17	0.4–1.4	
**AFP (ng/ml)**	Mean ± SD	14.94 ± 2.29	12.11 ± 2.05	0.000
	Range	10.5–22	9–20	
**HbA1c (%)**	Mean ± SD	5.73 ± 0.23	5.40 ± 0.24	0.000
	Range	5–6	5–6	

**Fig. 2:**
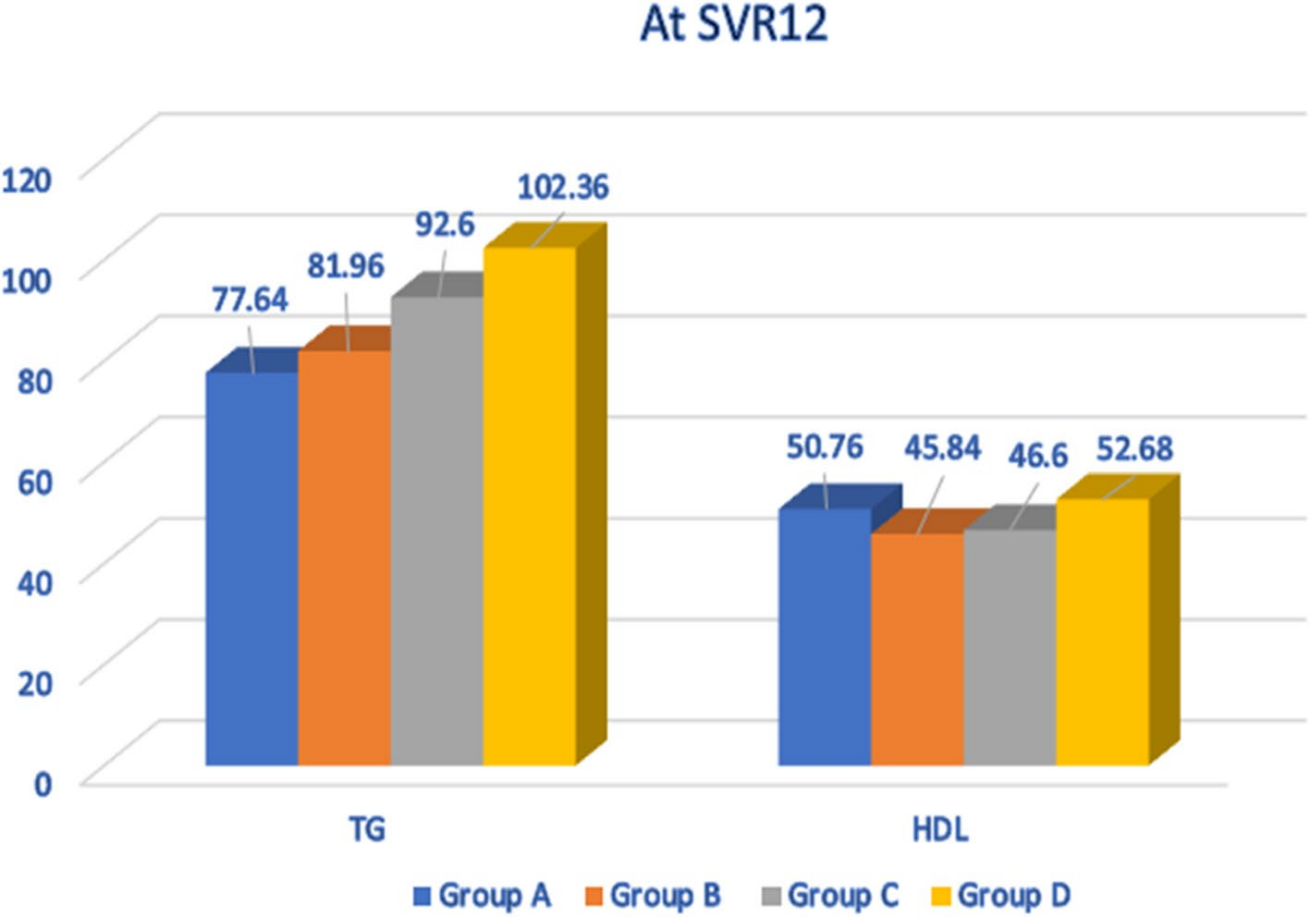
Comparison between all the patients in this study regarding the lipid profile before treatment and at SVR12

**Fig. 3:**
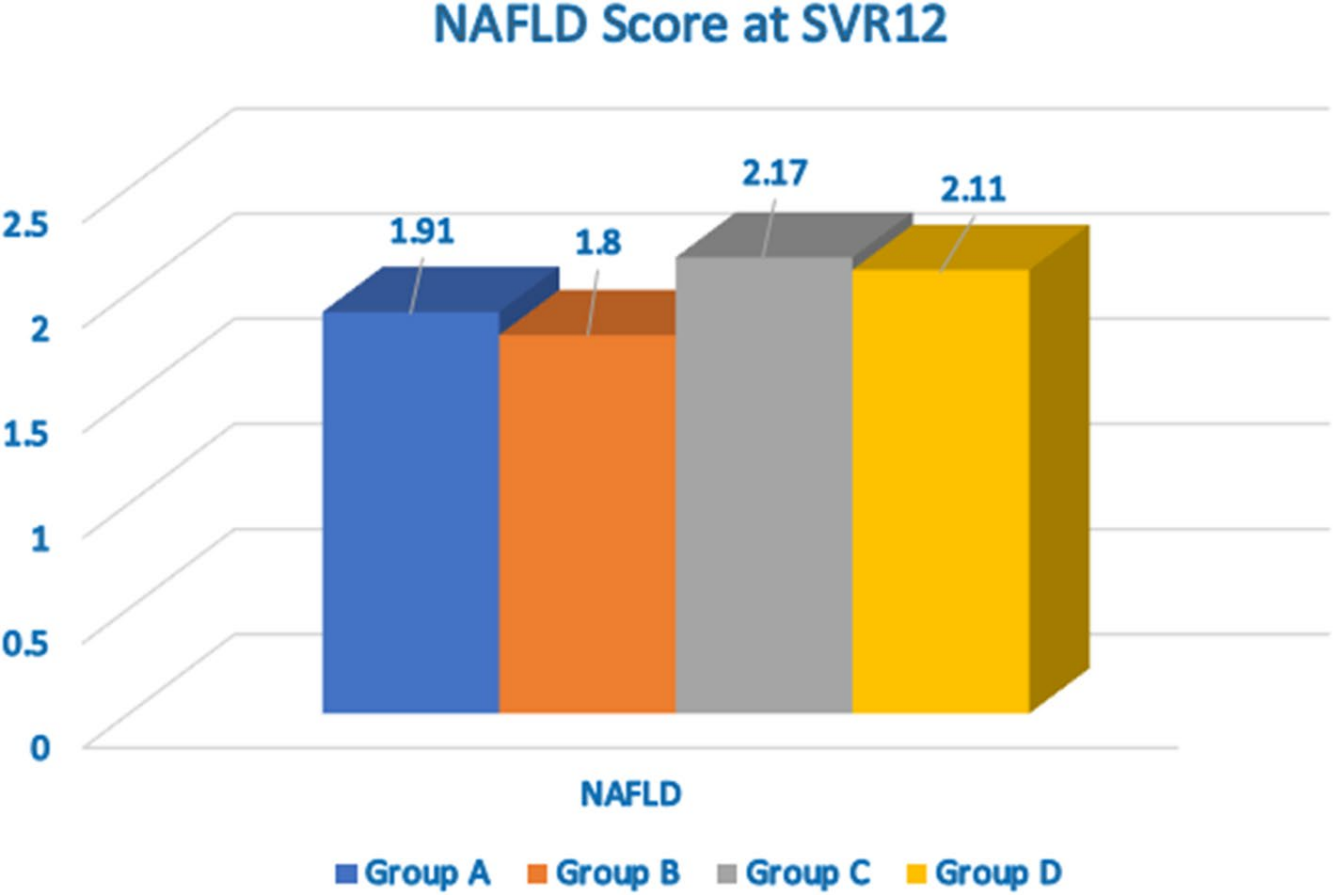
Comparison between the studied groups regarding serum TG and HDL levels at SVR12

The mean serum TG was significantly decreased from 101.68± 29.85 to 88.64±26.53 from baseline to SVR12 (Table 2, Fig. 2). The mean TG was different across the studied groups at SVR12, where the highest was in group D 102.36 ± 24.24 and the lowest was in group A 77.64 ± 25.37 (Fig. 3). The mean serum cholesterol was significantly decreased on comparing all the patients before starting treatment, and at SVR-12, the four groups were similar in their SVR12 cholesterol levels (Fig. 2). The mean HDL was significantly increased from 36.15±9.63 at baseline to 48.97 ±9.86 at SVR12 (Figs. 2 and 3, and Table 2). Moreover, there was a difference in HDL level between the four studied groups at SVR12, where the highest was in group D with a mean of 52.68 ±7.78 and the lowest was in group B with a mean of 45.84 ± 9.85 (Figs. 2 and 3). The mean LDL was significantly increased from 81.79±21.43 to 88.09 ±21.80, from baseline to SVR12, while there was no difference between the four groups at SVR-12 (Fig. 2).

**Fig. 4:**
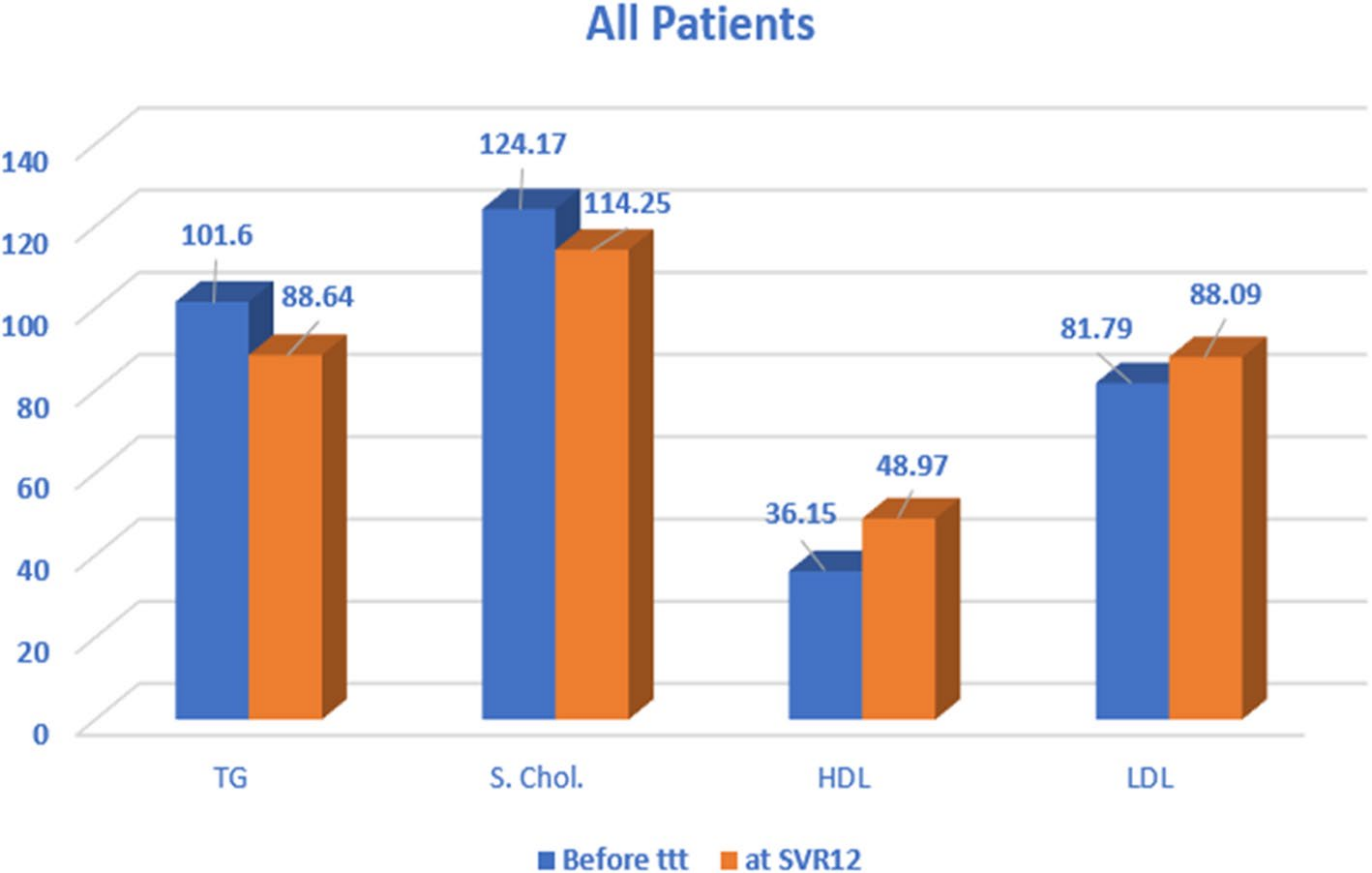
Comparison between the studied groups regarding NAFLD score at SVR12

**Fig. 5:**
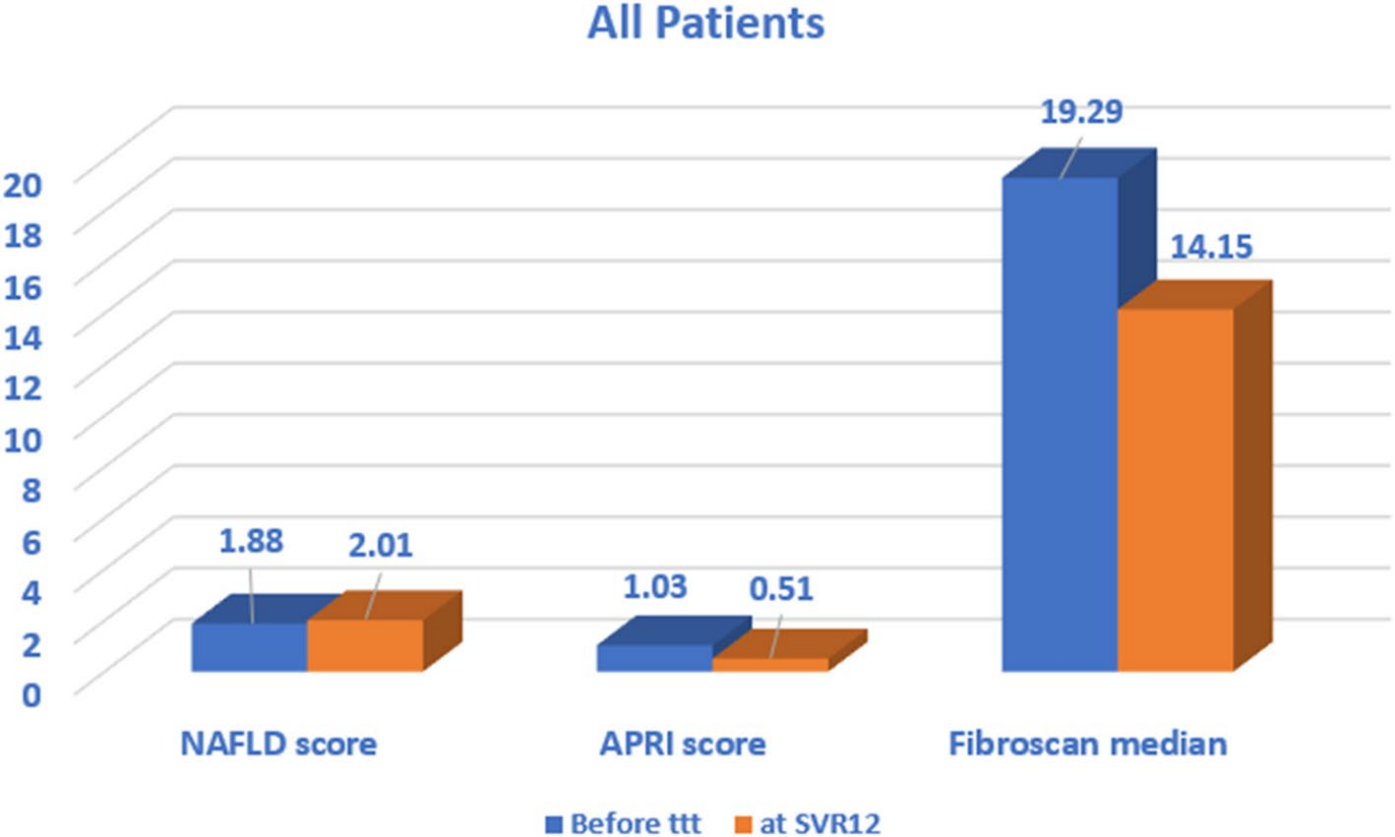
Comparison between all patients in this study regarding NAFLD and APRI scores and FibroScan median before treatment and at SVR12

APRI score mean was significantly decreased, while NAFLD score (Fig. 4) was increased from baseline to SVR12 (Fig. 5).

There was no difference in the FibroScan score, on comparing all the patients, before starting treatment, and at SVR 12. However, FibroScan median decreased from 19.29± 6.97 kPa at baseline to 14.15±6.48 kPa at SVR12. The FibroScan median at SVR12 was similar across the four groups (Fig. 5).

BMI mean decreased from 28.31 ± 1.53 at baseline to 28.07 ± 1.52 at SVR12 (Table 1). In addition, HbA1C level mean decreased from 5.73±0.23 at baseline to 5.40 ± 0.24 at SVR12 (Table 2). There was also a difference in HbA1C level across the studied groups at SVR12, where the highest level was in group D with a mean of 5.52 ± 0.23 while the lowest level was in group B with a mean of 5.34 ± 0.25.

## Discussion

After the high success of the combination regimens across the various studies and treatment centers, the AASLD guidelines issued a major update in November 2019 regarding HCV management [9]. In addition, the WHO plan to eliminate HCV globally by 2030 is working with high efficacy [16]. The HCV burden in Egypt is high. A study in our Ain Shams University published recently showed that the prevalence of HCV antibodies in healthcare workers was 8%, while among the attending patients was 19.8%. The highest prevalence was among patients > 50 years of age or living outside Cairo [17].

All patients in our study achieved SVR-12. This success rate is similar to many studies done after the introduction of DAAs [18–20]. Our study population is selected with a normal baseline metabolic profile, despite being chronically infected by HCV. Thus, any change in their metabolic profile post-therapy is attributed to the effect of medications and the HCV cure.

Our study shows that the efficacy of the different DAAs is comparable. The achievement of viral elimination and SVR was associated with an improvement in the blood and metabolic indices, along with an improvement in the prognostic scores as the APRI and the NAFLD scores. Moreover, the SVR is associated with a better transient elastography parameters posttreatment, indicating stabilization of the patient’s condition, and possible regression of the liver fibrosis stage.

The use of APRI and FIB-4 scores was compared to transient elastography. It was found that the combination of APRI/FIB-4 highly predicts the significant fibrosis F3/F4, while the FIB-4 alone highly predicts the cirrhosis F4 [14]. In our study, the APRI score was decreased, and NAFLD score was increased after reaching SVR-12 weeks.

The elevation in the lipid profile especially LDL could be explained by HCV nonstructural protein 5A activation of the sterol regulatory element-binding protein-1c (SREBP-1c), responsible for the activation of the lipogenic genes [21].

HCV is known to cause steatosis generally; however, the severity of steatosis is related to the viral genotype. Genotype 3 is most commonly associated with an increase in the steatosis severity, as the phenylalanine residue (position 164) of the HCV core particle causes an enhanced accumulation of lipid droplets in the hepatocytes [22].

In addition, HCV-associated steatosis could be caused by host factors as BMI, uncontrolled blood sugar, diabetes, and hyperlipidemia. Moreover, these host factors could participate in the increased progression to fibrosis and cirrhosis [23]. In our study, the fasting blood sugar and BMI were decreased upon achieving SVR.

In a previous Egyptian study using the regimen daclatasvir-sofosbuvir for 3 months, the total cholesterol was increased upon achieving SVR-12 and SVR-24, along with an increase in the LDL levels, but the triglycerides remained unchanged. This could be attributed to the baseline metabolic state of their cohort, as two-thirds of the included patients had a hyperlipidemic state prior to treatment [4]. Another study, in our Ain Shams virology treatment center, done on HCV-infected patients with decreased eGFR (of 30 ml/min or less), they found that after achieving SVR, the triglycerides levels, and HbA1C, were decreased, while the total cholesterol, LDL, and HDL levels were increased [18].

In our study (as shown in Fig. 2), we chose patients with normal metabolic profile and excluded the hyperlipidemic and the diabetic HCV-infected patients. We found that the decline upon achieving SVR-12 weeks was in the levels of HBA1C, the fasting blood glucose, the triglycerides, and the total cholesterol, while the level of HDL was elevated. This indicates an improvement in their metabolic profile; however, their LDL levels were increased. Our results agree with Gitto et al., where they observed worsening of the cholesterol, and LDL level, along with improved HDL after DAAs treatment, on a cohort of 100 of treated HCV patients [6].

Hashimoto et al. explained this disturbance in LDL by the deregulation of the lipid metabolism with subsequent dropping of the lipid droplet production and rebound elevation in the circulating LDL following the rapid viral suppression by the DAAs [24]. In addition, it is proved that the inflammatory state does not resolve completely even after the virus is completely eliminated by the DAAs [25]. Residual histological steatosis post-SVR could be detected on short-term follow-ups, despite the normalization of the liver enzymes [5].

It is controversial if there is a fibrosis regression post SVR-12; however, most of the fibrosis non-invasive parameters tend to decrease after cure of the viral load. It is shown that liver stiffness is influenced not only by the degree of liver fibrosis but also by necro-inflammatory activity [20]. This could explain the small decline in the transient elastography parameters in our study. In our study, liver stiffness decreased over time, and there were significant differences between baseline and SVR-12 weeks among the studied groups.

## Conclusion

DAAs affect the metabolic profile of the treated HCV patients. Different regimens may have different effects on the metabolic profile and the liver stiffness in HCV-treated patients. Early DAAs are still used in Egypt with good efficacy and good metabolic outcomes.

## Data Availability

The datasets used and/or analyzed during the current study available from the corresponding author on reasonable request. All figures supplied are original figures.

## Abbreviations

AASLD: The American Association for the Study of Liver Diseases
ALT: Alanine transaminase
APRI score: AST to platelet ratio index score
AST: Aspartate aminotransferase
BMI: Body mass index
DAAs: Direct-acting antivirals
EASL: The European Association for the Study of the Liver
ELISA: The enzyme-linked immunosorbent assay
HBA1C: Hemoglobin A1C
HCV: Hepatitis C virus
HDL: High-density lipoprotein
HIV: Human immunodeficiency virus
kPa: Kilopascal
LDL: Low-density lipoprotein
LLOQ: Lower limit of quantitation
PCR: Polymerase chain reaction
PLT: Platelets
RNA: Ribonucleic acid
SVR: Sustained virological response
